# Temporal dynamics of freshwater planktonic parasites inferred using a DNA metabarcoding time-series

**DOI:** 10.1017/S0031182021001293

**Published:** 2021-11

**Authors:** Kingsly C. Beng, Justyna Wolinska, Elisabeth Funke, Silke Van den Wyngaert, Alena S. Gsell, Michael T. Monaghan

**Affiliations:** 1 Department of Ecosystem Research, Leibniz Institute of Freshwater Ecology and Inland Fisheries (IGB), Berlin, Germany; 2 Institut für Biologie, Freie Universität, Berlin, Germany; 3 Department of Experimental Limnology, Leibniz Institute of Freshwater Ecology and Inland Fisheries (IGB), Berlin, Germany; 4 Wasser Cluster Lunz, Biologische Station GmbH., Lunz am See, Austria; 5 Department of Aquatic Ecology, Netherlands Institute of Ecology (NIOO-KNAW), Wageningen, The Netherlands

**Keywords:** 18S rRNA gene, aquatic food web, Genbank, long-term monitoring, metabarcoding, Müggelsee, planktonic parasites

## Abstract

Parasites are important components of biodiversity and contributors to ecosystem functioning, but are often neglected in ecological studies. Most studies examine model parasite systems or single taxa, thus our understanding of community composition is lacking. Here, the seasonal and annual dynamics of parasites was quantified using a 5-year metabarcoding time-series of freshwater plankton, collected weekly. We first identified parasites in the dataset using literature searches of the taxonomic match and using sequence metadata from the National Center for Biotechnology Information (NCBI) nucleotide database. In total, 441 amplicon sequence variants (belonging to 18 phyla/clades) were classified as parasites. The four phyla/clades with the highest relative read abundance and richness were Chytridiomycota, Dinoflagellata, Oomycota and Perkinsozoa. Relative read abundance of total parasite taxa, Dinoflagellata and Perkinsozoa significantly varied with season and was highest in summer. Parasite richness varied significantly with season and year, and was generally lowest in spring. Each season had distinct parasite communities, and the difference between summer and winter communities was most pronounced. Combining DNA metabarcoding with searches of the literature and NCBI metadata allowed us to characterize parasite diversity and community dynamics and revealed the extent to which parasites contribute to the diversity of freshwater plankton communities.

## Graphical abstract

**Figure:**
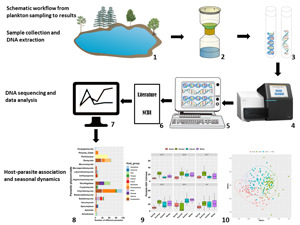

## Introduction

Parasites are important components of biodiversity and contributors to ecosystem functioning (Dobson *et al*., 2008; Hatcher *et al*., 2012; Frainer *et al*., 2018). Despite the recognized role of parasites in plankton community dynamics, and their tremendous diversity and species richness, most studies on planktonic parasites have either focused on specific parasite systems (Gordy *et al*., 2016; Grabner, 2017; Selbach *et al*., 2020) or covered a relatively short time period (Ortiz-Álvarez *et al*., 2018; Prati *et al*., 2020; Kagami *et al*., 2021), hindering our understanding of parasite diversity and its temporal dynamics. Although these previous studies provide important insights into the diversity of parasite taxa in freshwater ecosystems, none of them combines information on the abundance, diversity, composition and seasonal dynamics of parasite communities. This information is required for improved monitoring of ecosystem health, quantification of energy and nutrient flow, and development of more precise food web models.

Temperate freshwater environments experience temporal fluctuations in abiotic and biotic factors. These fluctuations can potentially influence the abundance, diversity and composition of different parasite communities across different seasons (Poulin, 2020; Martins *et al*., 2021). For example, seasonal changes in parasite abundance have been linked to temporal shifts in host abundance (Ibelings *et al*., 2011; Gsell *et al*., 2013), diet and feeding preferences (Prati *et al*., 2020). Quantifying seasonal changes in parasite communities is an important step towards understanding the role of parasites in the maintenance of biodiversity, and in the functioning and stability of temporally changing ecosystems (Combes, 1996; Hatcher *et al*., 2012).

Planktonic parasites are challenging to study, in part due to the high cost (in terms of money, time and labour) associated with sorting and identifying species from large-scale biodiversity inventories and a declining taxonomic expertise. The limited set of morphological features and the high level of cryptic (identical morphology) or pseudo-cryptic (fine scale morphological differences) diversity limits our ability to identify parasites, even with advanced microscopes (Fernández-Arévalo *et al*., 2020). For example, parasitic flagellated chytrid zoospores (infecting phytoplankton and amphibians) are often misidentified as bacterivorous flagellates (Lefèvre *et al*., 2007; Sime-Ngando *et al*., 2011; Kagami *et al*., 2012). Molecular methods detected eight microsporidian taxa that infect cladoceran *Daphnia*, including three taxa that had not been previously reported with conventional microscopic techniques (Weigl *et al*., 2012). In addition, parasites are typically small in size and/or ‘hidden’ within their hosts, and difficult to observe directly. Many parasites of plankton are either incompletely identified (e.g. genus or higher taxonomic levels) or exist only as environmental sequences (Lefèvre *et al*., 2008; Wolinska *et al*., 2009; Ortiz-Álvarez *et al*., 2018; Clarke *et al*., 2019). As such, some of these parasites are not included in curated reference databases. These explain why freshwater parasite diversity and community structure have been for a long time overlooked (Grabner, 2017; Garvetto *et al*., 2019; Selbach *et al*., 2020). This could lead to the underrepresentation of important trophic links in freshwater food webs (Lafferty *et al*., 2008; Amundsen *et al*., 2009) and an underestimation of the role of parasites in aquatic carbon and nutrient fluxes (Grami *et al*., 2011; Sánchez Barranco *et al*., 2020).

Advances in high-throughput sequencing techniques (e.g. DNA metabarcoding) have made it possible to detect cryptic and elusive parasites, including the undescribed life stages (e.g. cercariae and cysts) of more obscure species involved in disease transmission (Aivelo *et al*., 2015; Aivelo and Medlar, 2018; Greiman *et al*., 2018). However, standardized methods to identify parasites from metabarcoding datasets are lacking. We hypothesized that, in addition to the primary literature, metadata for DNA-sequence accessions in the National Center for Biotechnology Information (NCBI) could be an additional source of parasite and host information. This could potentially lead to a greater proportion of environmental sequences (i.e. DNA metabarcodes) being classified as parasites.

Here, we employed DNA metabarcoding, as well as searches of the literature and metadata from the NCBI nucleotide database, to quantify parasite biodiversity in a 5-year time-series of freshwater plankton from a temperate, polymictic eutrophic lake. We addressed the following questions: (i) Which planktonic parasitic taxa occur in the lake and what are their potential hosts? (ii) How do diversity, community composition and relative abundance of parasites vary across seasons and years?

## Materials and methods

### Study site and time-series data collection for plankton DNA metabarcoding

Müggelsee is a polymictic eutrophic lake located in the eastern suburbs of Berlin, Germany (52°25′–52°27′N, 13°37′–13°41′E). The lake has a mean depth of 4.9 m (max. 8 m) and a surface area of 7.3 km^2^ (Kohler and Hoeg, 2000). Every week between January 2015 and December 2019 (biweekly in January–February 2015), plankton were sampled from five locations in Müggelsee using a 5 L Friedinger sampler (Hydro-Bios Apparatebau GmbH, Kiel, Germany) and then combined to capture potential spatial heterogeneity in plankton communities. From this combined sample, a subsample (50–500 mL depending on plankton density as estimated by eye) was filtered through a glass fibre filter (Whatman GF/F, 25 mm diameter, 0.7 *μ*m pore size) using vacuum filtration at 200 mbar. All filters were freeze-dried (Alpha 1–4, Martin Christ Gefriertrocknungsanlagen GmbH, Osterode am Harz, Germany) for 8 h at −45°C and then stored at −20°C until DNA extraction.

### DNA extraction, PCR, library preparation and sequencing

Filters were placed into 2 mL Eppendorf tubes containing one stainless steel bead (5 mm diameter; Qiagen GmbH, Hilden, Germany), covered with TissueLyser (Qiagen) and shaken three times for 90 s at 30 Hz using a mixer mill MM301 (Retsch GmbH, Haan, Germany). The tubes were then given a short spin to collect all material in a pellet. DNA was extracted from the whole pulverized filter using the NucleoSpin Plant II extraction kit (Machery-Nagel, Düren, Germany) following the manufacturer's protocol. Extracted DNA was stored in TE buffer at −20°C until further processing.

All samples were amplified using the primers TAReuk454FWD1: CCAGCASCYGCGGTAATTCC and TAReukREV3: ACTTTCGTTCTTGATYRA (Stoeck *et al*., 2010) that target the V4 region of the 18S rRNA gene of eukaryotes. For all PCR reactions, template DNA (10 ng) was combined with 5 *μ*L reaction buffer (Herculase II Fusion DNA Polymerase, Agilent Technologies, Santa Clara, CA, USA), 0.5 *μ*L dNTP mixture (Agilent), 1 *μ*L of each primer, 0.4 *μ*L proof-reading polymerase (Herculase II Fusion DNA Polymerase, Agilent), 0.25 *μ*L 100 mm MgSO₄ (New England Biolabs, Ipswich, MA, USA) and RO-filtered water for a total reaction volume of 25 *μ*L. PCR (95°C for 30 s; 30 cycles of 95°C for 30 s, 45°C for 30 s, 72°C for 30 s; 72°C for 5 min) products were cleaned using a magnetic bead protocol (Agencourt AMPure XP, Beckman Coulter, Indianapolis, IN, USA) following the manufacturer's instructions. DNA concentration was measured using a Quantus fluorometer and the QuantiFluor^®^ dsDNA System (Promega, Madison, WI, USA), and all PCR products were normalized to a concentration of 5 ng *μ*L^−1^. A second PCR reaction (Indexing PCR) added unique 12-bp inline sequence barcodes (Nextera Index Kit, Illumina, San Diego, CA, USA) to each sample. The Indexing PCR was performed using 10 *μ*L of target PCR product as template mixed with 5 *μ*L reaction buffer (Herculase II Fusion DNA Polymerase, Agilent), 0.25 *μ*L dNTP mixture (Agilent), 0.625 *μ*L of each Index Primer P5 and P7 (Nextera Index Kit, Illumina), 0.25 *μ*L proof-reading polymerase (Herculase II Fusion DNA Polymerase, Agilent), 1 *μ*L DMSO and RO-filtered water for a total reaction volume of 25 *μ*L. PCR (95°C for 2 min; 8 cycles of 95°C for 20 s, 52°C for 30 s, 72°C for 30 s; 72°C for 3 min) products were purified twice and quantified as above. All samples were then pooled in equimolar amounts and sequenced on an Illumina MisSeq using a v3 sequencing kit (600 cycles). Sequencing was performed in three separate runs corresponding to samples collected between 2015 and 2017 (run 1, 125 samples), in 2018 (run 2, 51 samples) and in 2019 (run 3, 56 samples). Three negative controls (one *per se*quencing run) were included as part of all PCR reactions and were sequenced in the same run as the regular samples. Raw sequence data (fastq.gz files) are available at the Sequence Read Archive (BioProject accession number PRJNA526363).

### Bioinformatics analysis

Raw sequence data were analysed using the DADA2 pipeline (Callahan *et al*., 2016) in R (R Core Team, 2021). The complete R script is provided as a supplementary file (R-script.txt). Briefly, forward and reverse reads were trimmed of the primers (first 20 bases), reads were truncated (forward at position 260, reverse at position 200), and reads with a maximum number of expected errors ⩽2 were retained. Reads were then dereplicated and merged, and chimeras were removed *de novo*. Taxonomic assignment of amplicon sequence variants (ASVs) (Callahan *et al*., 2016) was performed using the assignTaxonomy function with default parameters in DADA2, and the Protist Ribosomal Reference (PR2 version 4.12.0) database (Guillou *et al*., 2013).

### Identification of potential parasite ASVs and their potential hosts

We used a combination of search criteria and methods to identify parasite ASVs (Table 1). In summary, higher taxonomic groups with known parasitic species were preselected. For ASVs that were assigned to species level within these taxonomic groups, we used both published literature (searching Google Scholar and Web of Science with the full binomial name, last search was performed on 5 March 2021) and associated metadata for the species in the NCBI nucleotide database to link the species or sequence to a host. For ASVs that were assigned to genus or to a higher taxonomic level, we used each ASV as a query to perform blastn searches against the NCBI nucleotide database (Benson *et al*., 2013). We then selected the top 10 matches to the query and checked their associated metadata in numerical order (from highest to lowest score). If the metadata of the first match contained information on one or more hosts, we recorded the host names or taxa. If host information was not provided, we proceeded to the second match, and repeated the procedure until host information was found. If none of the top 10 matches had host information, we classified the ASV as non-parasitic.

**Table 1.** Protocol for parasite identification using published literature and the NCBI nucleotide database

Step	Action	Remarks/assumptions
1	We preselected taxonomic groups with known parasitic species	Based on published literature (Ortiz-Álvarez *et al*., 2018)
2	Within these preselected groups, we selected all ASVs that were assigned to species-level	We assumed these assignments are correct
3	Species names were used to perform a literature search to determine whether or not an ASV was parasitic	If a species is parasitic, information about its host should be available in the literature
4	When host information was not found, we used the sequence of each ASV as a query to perform blastn searches against the GenBank database (Benson *et al*., 2013). We then selected the top 10 matches to the query and checked their associated metadata in numerical order (from highest to lowest score). If the metadata of the first match contained information on one or multiple hosts, we recorded the host names or taxa. If the host information was not provided, we proceeded to the second, third … and 10th match	The top 10 matches were only considered if Query Cover = 100% and Per cent identity ⩾90%. We checked the *E*-values of 44 (10%) random matches and they were all very low, ranging from 0.0 to 4 × 10^−57^ (Sheet 2, Supplementary Dataset 1)
5	For ASVs that were assigned to genus or higher taxonomic level using assignTaxonomy, step 4 was used	The top 10 matches were only considered if Query Cover = 100% and Per cent identity ⩾90%
6	If the top 10 matches had no host information, but had species names, we used step 3. Otherwise, we classified the ASV as non-parasitic	The taxonomic database might not be up-to-date as new records are, in most cases, first archived in NCBI then in other databases
7	Exceptionally, we considered all Perkinsozoa ASVs as parasites	Published literature refer to these groups as ‘exclusively’ parasitic (Mangot *et al*., 2011; Chambouvet *et al*., 2015; Jeon and Park, 2020)

### Parasite relative read abundance and richness

The entire dataset with all ASVs was first converted from an abundance matrix (number of reads of each ASV in a sample) into a relative abundance matrix (proportion of total sequencing belonging to each ASV in a given sample) using the R package funrar (Grenié *et al*., 2017). The subset of parasite ASVs (as identified in 2.4) was used for all further analyses of seasonal abundance (relative number of reads) and richness (number of ASVs).

Two-way analysis of variance (ANOVA) and pairwise multiple comparison post-hoc tests (Tukey's honest significance, hereafter TukeyHSD) were used to determine whether season, year or their interaction (season×year) significantly influenced the relative read abundance and richness of parasite ASVs. ANOVA and TukeyHSD analyses were conducted for all parasites combined and separately for each of the four taxonomic groups that had the highest abundance according to sequencing read counts (Chytridiomycota, Dinoflagellata, Oomycota and Perkinsozoa; see Results). Seasons were defined as spring (March, April and May), summer (June, July and August), autumn (September, October and November) and winter (December, January and February).

### Parasite *β* diversity and community composition

The *β* diversity was computed for each year as multi-sample Sorensen and Simpson dissimilarity indices using the R package betapart (Baselga and Orme, 2012). These computations were done using 30 samples (number of samples ranged from 34 in 2015 to 56 in 2019) drawn at random for each year and resampled 100 times. The among-sample *β*-diversity (calculated using pairwise samples) was then decomposed into turnover (species replacement from one sampling time point to another) and nestedness (species gain/loss from one sampling time point to another).

Permutational multivariate ANOVA [PERMANOVA, R package vegan (Oksanen *et al*., 2019)] and pairwise multiple comparison [pairwise.adonis, R package Ecolutils (Salazar, 2020)] were used to test the influence of season, year and their interaction on the community composition of parasites. Non-metric multidimensional scaling (NMDS) with Bray–Curtis distance was used to visualize the similarity among samples based on ASV composition. The smaller the distance between two samples, the higher the similarity between their ASV assemblages. The Simper function, which discriminates species between two groups using Bray–Curtis dissimilarities, was used to determine the contribution of individual ASVs to the overall community dissimilarity (Oksanen *et al*., 2019). Simper performs pairwise comparisons of groups of samples (e.g. winter *vs* summer) and finds the average contributions of each species to the average overall Bray–Curtis dissimilarity (Oksanen *et al*., 2019).

## Results

**Fig. 1.**
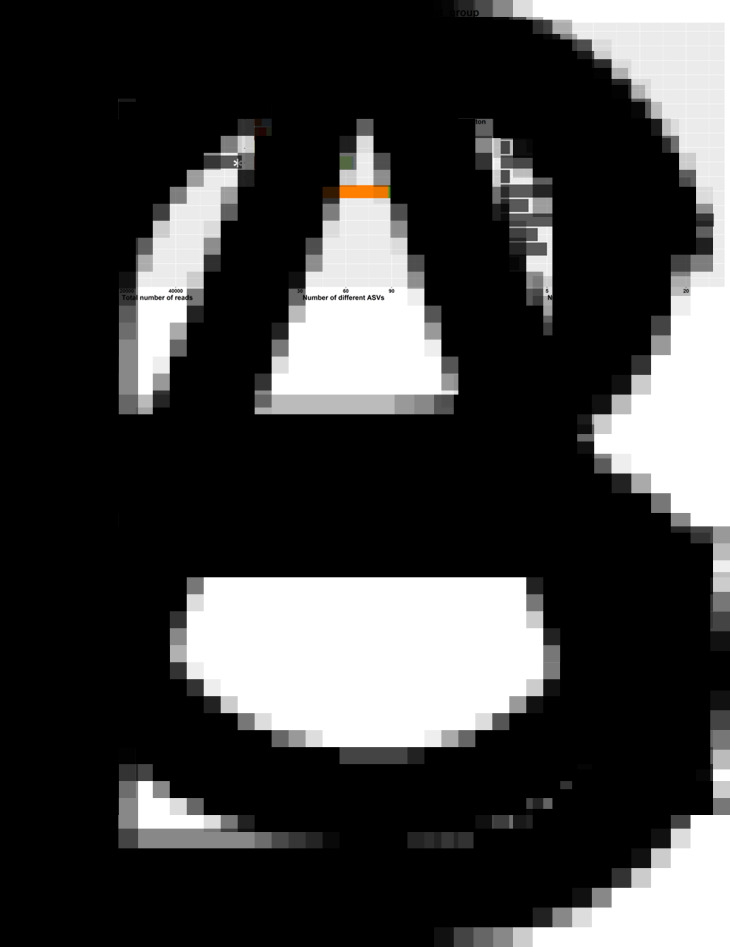
Total number of reads (A), number of different ASVs and their potential host taxa (B), and number of ASVs assigned to species level (C) in each parasitic phylum/clade. *The total number of reads shown was set at 65 000 to conveniently visualize the abundance of all parasitic phyla/clades but Dinoflagellata had 209 013 total reads.

A total of 12 004 135 raw reads were obtained across all years from three partial Miseq runs. Sixty per cent (7 168 910) of all reads passed filtering, denoising, merging and chimera-removal steps and resulted in 7128 ASVs in total. The negative controls contained 555 reads (34 ASVs, including six Chlorophyta, two Ciliophora, one Cryptophyta, two Fungi, two Metazoa, 17 Ochrophyta and four Streptophyta). None of the ASVs from these reads were classified as parasitic by our approach (see Methods section ‘Identification of potential parasite ASVs and their potential hosts’). ASVs that were present in only one sample (out of 232) were removed, resulting in a total of 3063 ASVs used for downstream analyses. Of these, 441 (14.4%) ASVs were identified as parasites, 107 based on literature searches, 281 based on NCBI metadata, and 53 from both literature and NCBI sources (Supplementary Dataset 1). These parasite ASVs were classified into 18 higher taxonomic groups (phyla/clades), with the four most read-abundant groups being Chytridiomycota, Dinoflagellata, Oomycota and Perkinsozoa (Fig. 1A). These same four groups had the highest number of ASVs (Fig. 1B; Chytridiomycota 117, Oomycota 69, Dinoflagellata 65 ASVs and Perkinsozoa 53 ASVs). Of the 441 ASVs, 118 could be assigned to 54 putative species whereas the rest were assigned to genus or higher taxonomic levels (Supplementary Dataset 1). Taxa with the highest number of ASVs identified to species level were Basidiomycota (23), Chytridiomycota (17), Oomycota (7) and Dinoflagellata (6) (Fig. 1C).

The Chytridiomycota ASVs we identified were reported to be parasites of phytoplankton, zooplankton, amphibians, fungi and plants. The most abundant species were *Rhizophydium littoreum*, a parasite of the yellow rock crab [*Cancer anthonyi*, Shields (1990)]; *Zygophlyctis planktonica*, a parasite of the diatom, *Synedra sp.* and *Zygophlyctis melosirae*, a parasite of the diatom genus, *Aulacoseira* (Seto *et al*., 2020); *Gaertneriomyces semiglobifer*, a parasite of the moth-killing fungus *Entomophaga maimaiga* (Hajek *et al*., 2013); and *Dangeardia mamillata*, a parasite of the green alga, *Yamagishiella unicocca* (Van den Wyngaert *et al*., 2018).

Oomycota that we found were parasites of zooplankton, nematodes, plants, phytoplankton, fish and amphibians. The most abundant species was *Chlamydomyzium dictyuchoides*, a parasite of nematodes (Beakes *et al*., 2014).

The Dinoflagellata ASVs were parasites of fish, sponges, zooplankton and amphibians. The most abundant species were *Chytriodinium roseum*, a parasite of euphausiid eggs (*Meganyctiphanes norvegica*, Gómez-Gutiérrez *et al*., 2009); *Naiadinium polonicum*, which matched a sequence isolated from the gut content of fish (*Ctenochaetus striatus*, Genbank accession MN188877); *Asulcocephalium miricentonis*, which matched a symbiotic dinoflagellate sequence associated with the sponge, *Baikalospongia recta* (Genbank accession, FJ823473); *Ceratium furcoides*, which matched a sequence isolated from swab samples of the amphibian, *Thoropa taophora* (Belasen *et al*., 2019).

The hosts of Perkinsozoa parasites were classified as ‘Various’ based on literature and they include shellfish, fish, dinoflagellates, cryptophytes and amphibians (Chambouvet *et al*., 2015; Jeon and Park, 2020).

**Fig. 2.**
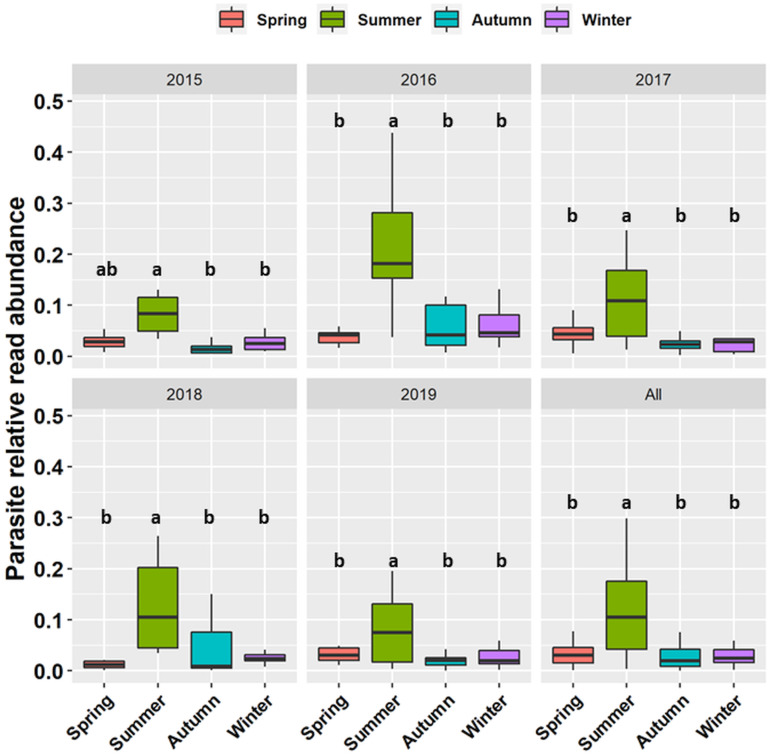
Seasonal differences in the read abundance of the 441 parasite ASVs relative to the total number of reads in the entire metabarcoding dataset. Different letters (a, b) represent significant differences while similar letters (a, a) represent non-significant differences. Outliers have been hidden by setting outlier.shape = NA

**Fig. 3.**
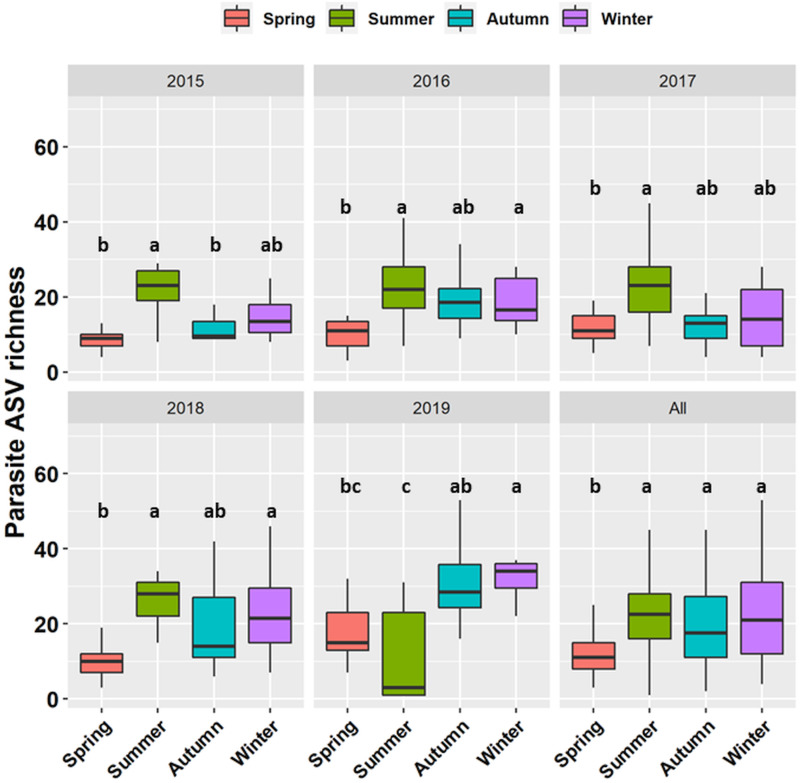
Seasonal differences in parasite richness (observed number of parasite ASVs). Different letters (a, b) represent significant differences while similar letters (a, a) represent non-significant differences. Outliers have been hidden by setting outlier.shape = NA

The relative abundance and richness of all parasites (i.e. all 441 ASVs, regardless of taxonomic group) changed significantly with season and year (Table 2). Parasite sequencing reads were usually 5–20% of total reads (range 0.5–60%) and this percentage was typically highest in summer (Fig. 2). Patterns of ASV richness differed across years but richness was typically lowest in spring (Fig. 3). Analysed separately, the relative read abundance of Dinoflagellata and Perkinsea varied seasonally but Chytridiomycota and Oomycota did not (Table 2), with both Dinoflagellata and Perkinsozoa having higher abundance in summer (Fig. S1, Supporting information). The interaction between season and year was significant for Chytridiomycota and Dinoflagellata relative read abundance (Table 2). Richness of Dinoflagellata, Perkinsozoa and Oomycota varied seasonally (Table 2), being highest in summer (Fig. S2, Supporting information).

**Table 2.** Analysis of variance table showing the effects of season, year and their interaction (season × year) on parasite relative read abundance and richness

Level			Parasite ASV read abundance		Parasite ASV richness	
		D.F.	*F* value	Pr(>*F*)	*F* value	Pr(>*F*)
Total community	Season	3	33.09	<2 × 10^−16^***	14.53	1.21 × 10^−08^***
	Year	4	9.29	6.09 × 10^−07^***	5.45	0.00034***
	Season × Year	12	1.50	0.126 ns	5.06	2.09 × 10^−07^***
Chytridiomycota	Season	3	0.110	0.954 ns	2.41	0.067 ns
	Year	4	5.53	0.000293***	5.71	0.00022***
	Season × Year	12	2.74	0.001777**	1.80	0.04960*
Dinoflagellata	Season	3	27.96	2.82 × 10^−15^***	23.07	5.77 × 10^−13^***
	Year	4	7.38	1.37 × 10^−05^***	1.91	0.108
	Season × Year	12	2.99	0.000697***	3.37	0.00016***
Oomycota	Season	3	1.27	0.285 ns	14.06	2.15 × 10^−08^***
	Year	4	1.25	0.289 ns	3.22	0.0136*
	Season × Year	12	0.74	0.707 ns	3.59	6.68 × 10^−05^***
Perkinsozoa	Season	3	16.98	6.38 × 10^−10^***	33.73	<2 × 10^−16^***
	Year	4	0.27	0.896 ns	1.96	0.100
	Season × Year	12	0.63	0.809 ns	3.33	0.000182***

Significance codes: *P < 0.05; **P < 0.01; ***P < 0.001.

**Fig. 4.**
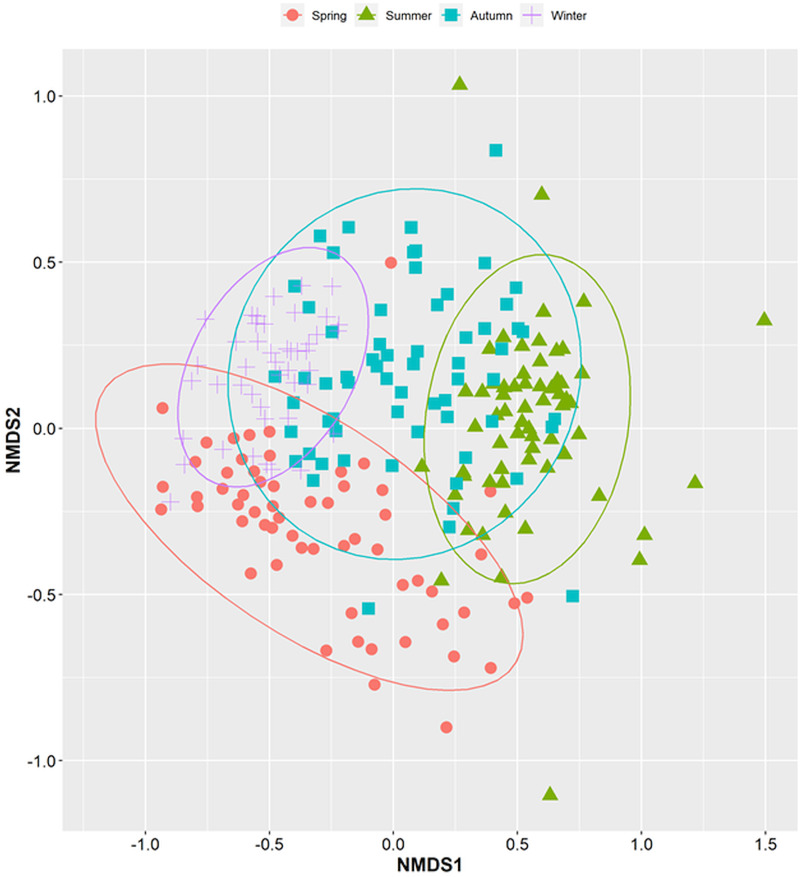
Non-metric multidimensional scaling (NMDS) of parasite community structure across four seasons (spring, summer, autumn and winter).

The community composition of parasites changed significantly with season and year (Table 3). The NMDS analysis revealed there was no overlap between summer and winter communities, but considerable overlap of autumn with all other seasons and of spring with both winter and autumn (Fig. 4). Our analysis of species turnover found that species replacement rather than nestedness was the primary driver of turnover (Fig. S3, Supporting information).

**Table 3.** Permutational analysis of variance and multiple pairwise comparison results showing the effects of season, year and their interaction (season × year) on parasite community structure

	D.F.	SS	*R*^2^	*F*	Pr(>*F*)
Season	3	15.20	0.15	15.17	0.001***
Year	4	4.15	0.04	3.11	0.001***
Season × Year	12	8.57	0.09	2.13	0.001***
Residuals	212	70.76	0.72		
Total	231	98.69	1.00		
Combination	SS	*F* model	*R*^2^	*P* value	*P* value corrected
Autumn <-> spring	2.78	6.86	0.05	0.0009	0.0009***
Autumn <-> summer	4.06	10.67	0.08	0.0009	0.0009***
Autumn <-> winter	3.82	10.45	0.09	0.0009	0.0009***
Spring <-> summer	7.49	20.45	0.15	0.0009	0.0009***
Spring <-> winter	2.77	7.97	0.07	0.0009	0.0009***
Summer <-> winter	9.50	29.50	0.21	0.0009	0.0009***

Significance code: ***P < 0.001.

The three main contributors to the spring-autumn average between-group dissimilarity were ASV0043 [Thoracosphaeraceae sp. (Dinoflagellata): 13.1%], ASV0062 [*Dissodinium pseudolunula* (Dinoflagellata): 8.8%] and ASV0014 [*N. polonicum* (Dinoflagellata): 7.1%]; winter-autumn were ASV0043 (Thoracosphaeraceae sp.: 13.1%), ASV0014 (*N. polonicum*: 6.5%) and ASV0109 [Cystobasidiomycetes sp. (Basidiomycota): 6.2%]; winter-spring were ASV0043 [Thoracosphaeraceae sp. (Dinoflagellata): 17.4%], ASV0062 (*D. pseudolunula*: 8.1%) and ASV0109 (Cystobasidiomycetes sp.: 6.8%); spring-summer were ASV0014 (*N. polonicum*: 21.4%), ASV0043 (Thoracosphaeraceae sp.: 7.9%), ASV0044 (*N. polonicum*: 6.0%); summer-autumn were ASV0014 (*N. polonicum*: 22.6%), ASV0044 (*N. polonicum*: 6.4%) and ASV0078 [*A. miricentonis* (Dinoflagellata): 3.7%]; and winter-summer were ASV0014 (*N. polonicum*: 20.1%), ASV0043 (Thoracosphaeraceae sp.: 8.5%) and ASV0044 (*N. polonicum*: 5.7%) (Supplementary Dataset 2).

## Discussion

Comprehensive information on the temporal patterns of abundance, richness and community composition is lacking for planktonic parasites in freshwater ecosystems (Grabner, 2017; Berkhout *et al*., 2020; Selbach *et al*., 2020). Over a period of 5 years, sampling weekly in all but a few months that were sampled biweekly, we detected 441 parasite ASVs belonging to 18 parasitic phyla/clades. Our finding that 14.4% of all ASVs in the lake were parasitic was similar to a previous survey based on single samples from 227 lakes which found 18% of all planktonic OTUs to be parasitic (Ortiz-Álvarez *et al*., 2018). That study was carried out in summer, when we also recorded the highest diversity and relative read abundance of parasites. Unlike our study, they classified OTUs as parasitic based on their membership in broad taxonomic groups without confirming the host of each individual OTU [e.g. all Chytridiomycota OTUs were classified as parasites (Ortiz-Álvarez *et al*., 2018) although they are known to be saprophytic as well (Frenken *et al*., 2017)]. As such, our results are more conservative, in that parasitic groups may have been removed from our analysis because no host information was available for that species, or for that sequence and closely related sequences.

Literature and NCBI searches revealed that the parasite ASVs we observed potentially interact with a wide range of hosts. Although some of these hosts are not known to occur in Müggelsee, they occur in other lakes. It is therefore possible that these ASVs still have the same trophic strategy but different hosts. For example, sponges are a potential host of the parasitic taxon Dinophyceae which had the highest read abundance in this study. Sponges account for 44% of the benthic biomass of Lake Baikal (Pile *et al*., 1997; Kenny *et al*., 2019) and are commonly infected by parasites (Zvereva *et al*., 2019; Lipko *et al*., 2020). Sponges are not known from Müggelsee, although another DNA metabarcoding study (M.T. Monaghan *et al*., unpublished data) uncovered sequences from *Spongilla lacustris* (Porifera, Spongillidae). Nonetheless, the lack of record suggests that abundance is very low and that Dinophyceae are likely parasites of other taxa in Müggelsee. We also found many parasitic taxa linked to hosts with terrestrial origins. For plants, it is possible that leaves, flowers and wood from terrestrial environments get transported into the lake by runoff or wind (Korosi *et al*., 2015). It can also be that these are parasites of freshwater macrophytes.

The four most abundant and diverse groups of parasites were chytrids, dinoflagellates, oomycetes and Perkinsozoa, and the four most frequent host taxon groups were phytoplankton, zooplankton, amphibians and fish. The fact that chytrids were the most abundant/diverse group is probably partly the result of bias arising from differential research interests across taxonomic groups and potential taxonomic biases in aquatic host–parasite research. For example, since the discovery of *Batrachochytrium dendrobatidis*, a chytrid responsible for mass mortality in amphibians, there has been a significant increase in chytrid research (Longcore *et al*., 1999; Fisher and Garner, 2020). The important role of chytrids in food webs has also attracted more research on their ecology and interaction with algal hosts (Kilias *et al*., 2020; Rasconi *et al*., 2020; Frenken *et al*., 2020*a*, *b*). Historically, the development was the other way around, first interest in the impact of chytrids on specific host algae [see research by Canter and Lund (60 s) and Van Donk (80 s–90 s)] which then evolved into the impact of chytrids on food webs and ecosystem functions (2000s). Nonetheless, it is increasingly recognized that chytrids are one of the most diverse and abundant taxonomic groups that occur in lake pelagic zones (e.g. Comeau *et al*., 2016; Wurzbacher *et al*., 2016).

The relative abundance, richness and community composition of parasites changed with season and year, corroborating previous findings that different parasites can occur, reproduce, interact or become abundant during different periods of the year (Ibelings *et al*., 2011; Alacid *et al*., 2017). There was no overall effect of season on the relative abundance of Chytridiomycota and Oomycota parasites, but there was an effect of year and a crossover interaction (significant interaction effect and non-significant main effects) for Chytridiomycota. This suggests that the effect of season on the relative abundance of Chytridiomycota changes depending on the year and may be linked to differences in both intra- and inter-annual dynamics of algal hosts, and abiotic conditions (Dakos *et al*., 2009; Gerphagnon *et al*., 2017). The lack of seasonal changes in relative read abundance of Oomycota may be explained by their observed wide range of hosts (Wolinska *et al*., 2008). For example, the same oomycete (based on sequence data) was reported from *Daphnia*, other cladocerans, fish and green algae (Wolinska *et al*., 2008).

Each of the four seasons considered had distinct parasite communities and the abundance and richness of the four most diverse and read-abundant parasitic taxonomic groups considered peaked in different seasons. This indicates that each season supports a unique parasite assemblage. The strong differences in compositional dissimilarity were mainly due to species turnover, where each individual season gains additional (unique) species not found in the other three seasons. Interactions among host life-history traits, environmental heterogeneity and seasonality might be driving these patterns (Berkhout *et al*., 2020; Verhaegen *et al*., 2020).

We have demonstrated the utility of reference sequence metadata for inferring the potential hosts of taxonomically diverse but unclassified parasite species and the urgent need to improve these databases. To the best of our knowledge, this study is the first to combine literature search and NCBI metadata to infer potential host–parasite links. NCBI is a valuable platform for biodiversity research, especially for taxonomic assignment of sequences from metabarcoding projects. Major limitations of our approach are the taxonomic resolution of the 18S rRNA gene and the incompleteness of the NCBI metadata. We might have misclassified some ASVs as parasitic and/or as non-parasitic simply because of the limited taxonomic resolution and/or species delimitation of the 18S marker that was used (Abouheif *et al*., 1998). This is possible for taxonomic groups with phylogenetically closely related parasitic and saprophytic species (e.g. Chytridiomycota and Oomycota). The ⩾90% identity threshold seems low for high confidence species assignment, but our aim was to also identify potential parasites that are currently described at genus or higher taxonomic levels or submitted as ‘uncultured’ sequences. A previous study investigating the spatial and temporal dynamics of zooplankton communities in marine and freshwater ports also sequenced the 18S rRNA gene (V4 region) and used 75–100% similarity threshold BLAST hits for taxonomic assignment (Chain *et al*., 2016). The metadata of some parasite entries do not contain host information. This is particularly true for entries from studies that focused on taxonomy or phylogenetic placement. For example, the chytrid, *Rhizophydium planktonicum* Canter emend., a parasite of the diatom, *Asterionella formosa* Hassall (Seto *et al*., 2017), has four NCBI records (Accessions: LC176296.1, LC176291.1, LC176286.1 and FJ799984.1) but the associated metadata does not contain host information. However, rather than assume general groups without assigning a host to each ASV as done in a previous study (Ortiz-Álvarez *et al*., 2018), we checked for host information in NCBI and in published literature for each ASV separately. Although this method is very efficient for well-known parasites and/or hosts, it is not the case for understudied parasites and/or hosts, and calls for an improved integration of reference sequences and metadata for parasites in the future and targeted sequencing of infected hosts to clarify the parasitic association. Before being able to fully harness the power of NCBI and other reference sequence databases for parasite research, we stress the importance of increasing the taxonomic identification and reference barcoding of parasites, especially in freshwater ecosystems.

## Data Availability

Nucleotide sequence data reported in this paper are available at the Sequence Read Archive (BioProject accession number PRJNA526363).

## Supplementary material

For supplementary material accompanying this paper visit https://doi.org/10.1017/S0031182021001293. click here to view supplementary material
