# Supplementary material for: Temporal dynamics of freshwater planktonic parasites inferred using a DNA metabarcoding time-series
Source: Parasitology. 2021 Jul 19;148(13):1602–11. doi: 10.1017/S0031182021001293 (PMC8573423; doi:10.1017/S0031182021001293)
Supplement: Supplementary file 1 [file S0031182021001293sup.zip › S0031182021001293sup004.pdf]

Temporal dynamics of freshwater planktonic parasites inferred using a DNA metabarcoding time series

Kingsly C. Beng[1], Justyna Wolinska[1,2], Elisabeth Funke[1], Silke Van den Wyngaert[3,4], Alena S. Gsell[5], Michael T. Monaghan[1,2]

[1]Department of Ecosystem Research, Leibniz Institute of Freshwater Ecology and Inland Fisheries (IGB), Berlin, Germany

[2]Institut für Biologie, Freie Universität Berlin, Germany

[3]Department of Experimental Limnology, Leibniz Institute of Freshwater Ecology and Inland Fisheries (IGB), Berlin, Germany

[4]Wasser Cluster Lunz, Biologische Station GmbH., Lunz am See, Austria

[5]Department of Aquatic Ecology, Netherlands Institute of Ecology (NIOO-KNAW), Wageningen, The Netherlands

*Corresponding author

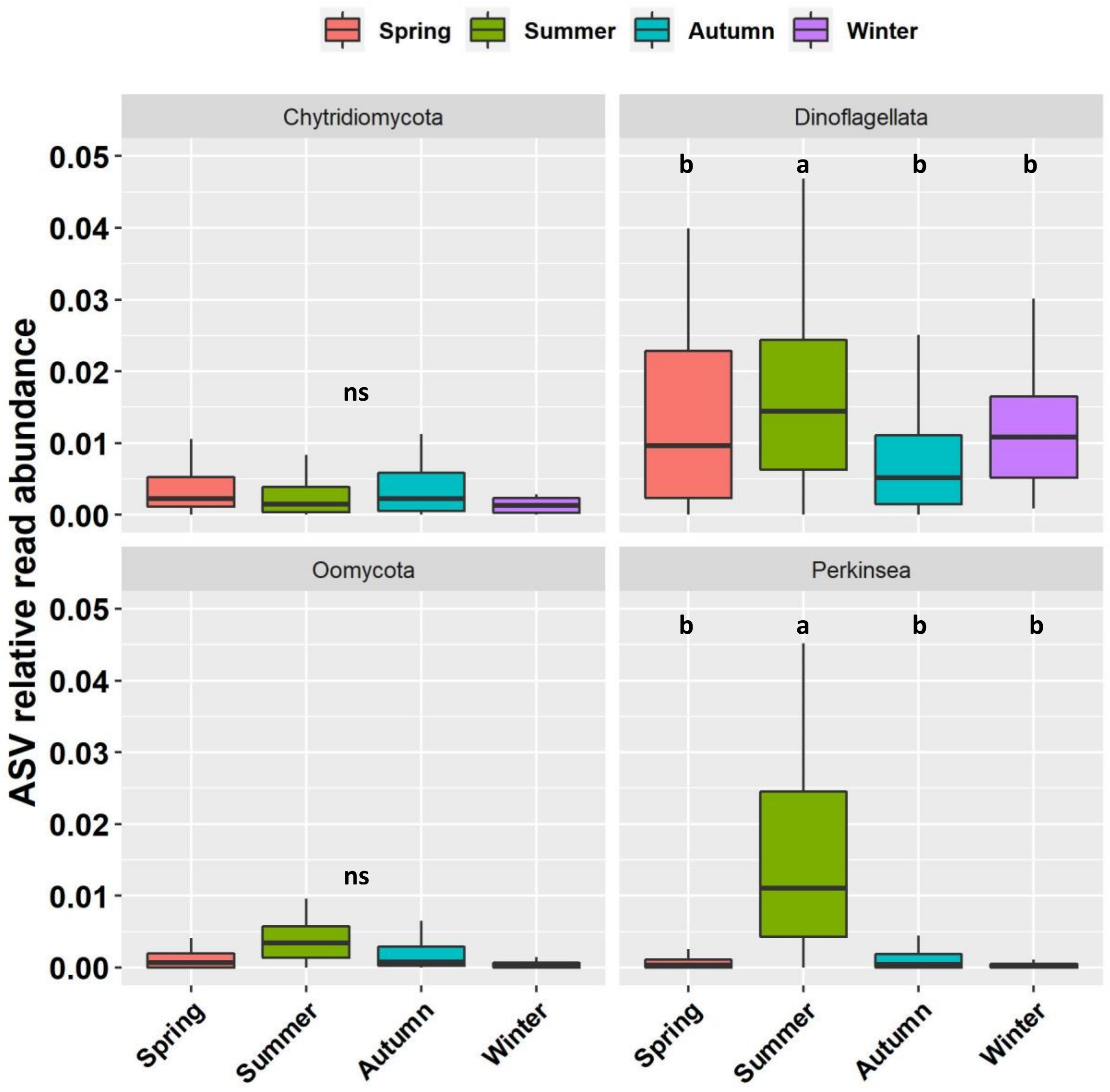

Figure S1. Seasonal differences in the abundance of parasitic ASV reads in each taxonomic group relative to the total number of reads in the entire metabarcoding dataset. Different letters (a, b) represent significant differences while similar letters (a, a) represent non-significant differences. ns = non-significant.

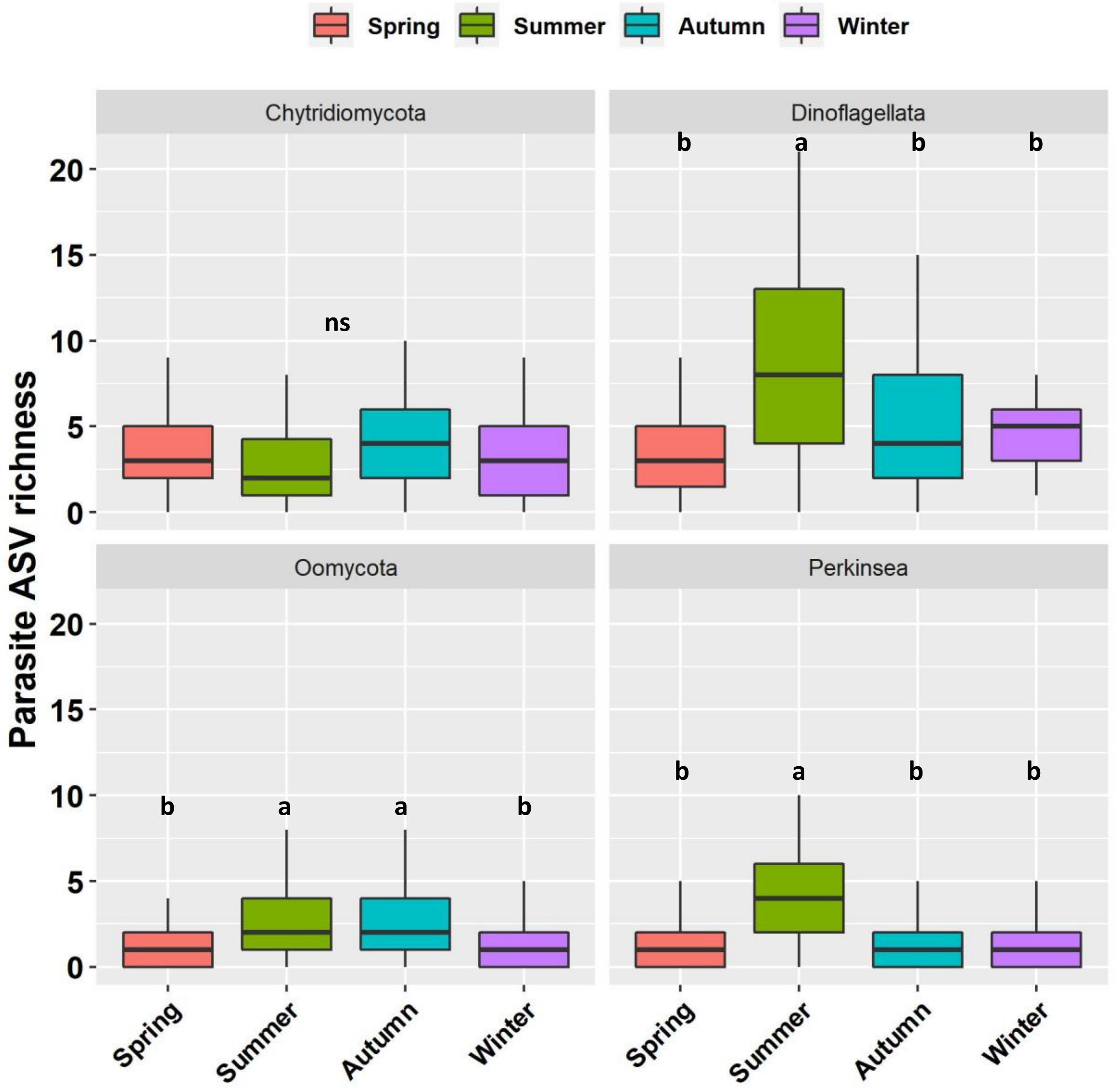

Figure S2. Seasonal differences in parasite ASV richness (observed number of parasitic ASVs) across taxonomic groups. Different letters (a, b) represent significant differences while similar letters (a, a) represent non-significant differences.

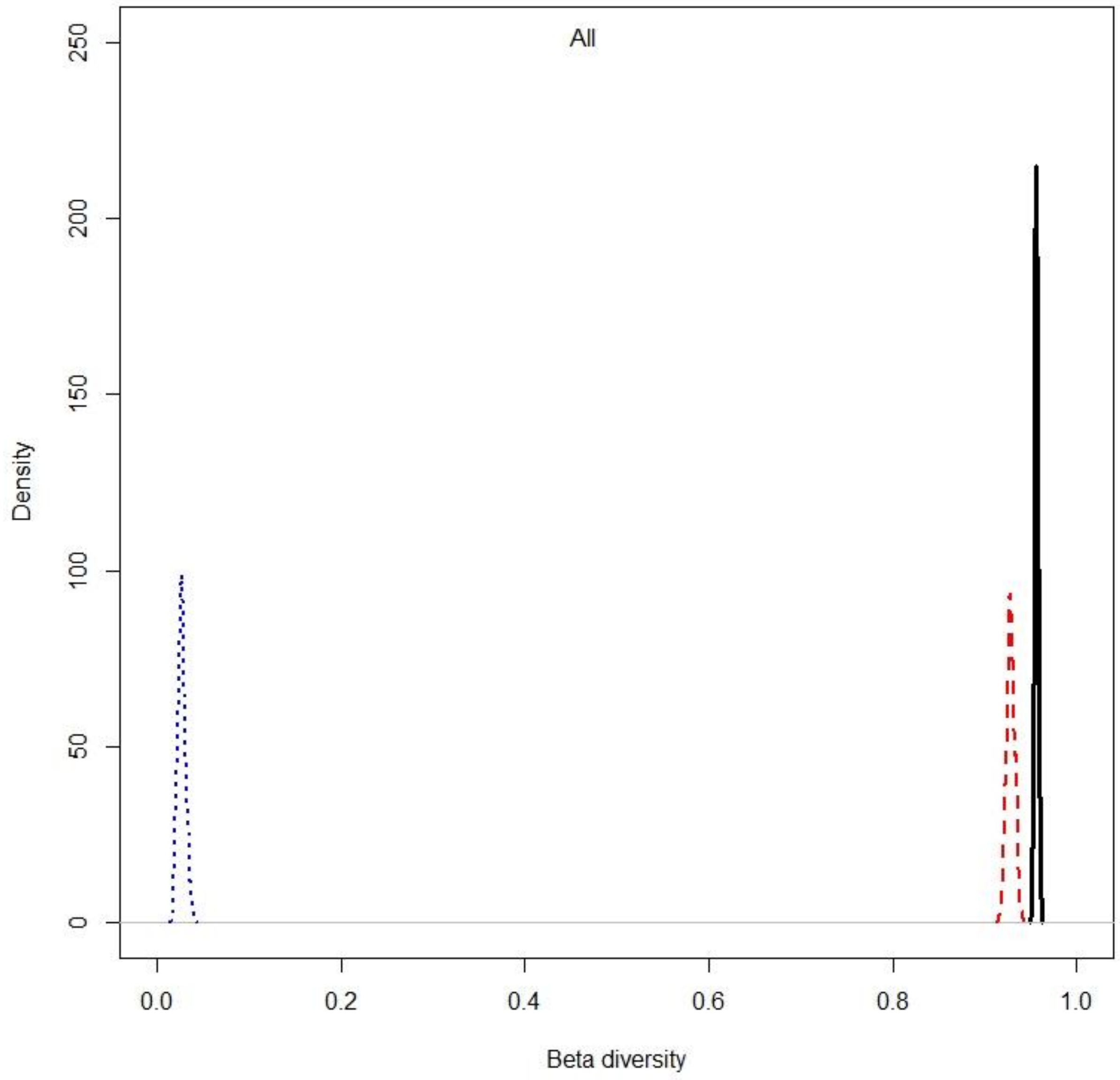

Figure S3. Parasite beta diversity decomposed into total (black smooth line), turnover (red broken line) and nestedness (blue dotted line) components. Beta diversity values range from 0 to 1, with 0 meaning low and 1 high beta diversity, respectively.
